# Effects of rumen-protected biotin on the growth performance, nitrogen utilization and blood parameters of yearling Liaoning cashmere doelings

**DOI:** 10.5713/ab.23.0078

**Published:** 2023-08-23

**Authors:** Haiying Liu, Ying Lin, Xuhui Chen, Guiqin Yang

**Affiliations:** 1 College of Animal Science and Veterinary Medicine, Shenyang Agricultural University, Shenyang 110866, China

**Keywords:** Growth Performance, Liaoning Cashmere Goats, Nitrogen Metabolism, Nutrient Digestion, Rumen-protected Biotin

## Abstract

**Objective:**

This study was conducted to investigate the effects of rumen-protected biotin (RPB) on growth performance, nutrient digestibility, nitrogen utilization and plasma biochemical parameters of Liaoning cashmere goats during the cashmere fiber growing period.

**Methods:**

Sixteen 6-month-old Liaoning cashmere twin-doelings (24.8±1.20 kg) were allocated to 2 diet groups that were individually *ad libitum* fed 30% concentrate and 70% forage diet (dry matter [DM]) by a paired experimental design. Goats of the control group were fed the basal diet, while goats belonging to the RPB group were fed the basal diet with 10 mg RPB/d per animal. The duration of the experiment was 16 weeks with two 8-week periods. Digestibility was determined at weeks 7 and 15, and other measures were taken every four weeks.

**Results:**

Compared with the control group, the average daily gain of the RPB group increased by 10.94% (p<0.05), and the intake of neutral detergent fiber was increased (p = 0.045). There were some increasing tendencies for the intake of DM, acid detergent fiber and ether extract (p = 0.070, 0.088, and 0.070, respectively). The intake and digestibility of N tended to increase (p = 0.062 and 0.093, respectively), while the N fecal excretion percentage of N intake was decreased (p = 0.093) in the RPB compared with the control group. N retention tended to increase (p = 0.084) with the addition of adding RPB to the diet. Plasma total protein was increased (p = 0.037), whereas the urea-N concentration was decreased (p = 0.049) in the RPB diet group compared with the control diet group. The levels of propionyl-CoA carboxylase (p<0.001) and methylmalonyl-CoA (p = 0.013) were increased in the RPB group.

**Conclusion:**

Supplementation of rumen-protected biotin in the diet of cashmere goats can enhance the utilization of N and improve daily weight gain during cashmere fiber growing period.

## INTRODUCTION

Biotin is a water-soluble vitamin containing sulfur that is an essential nutrient for the growth of animals [1]. It is covalently bound to a lysine residue of the carboxylase protein in methylcrotonyl-coenzyme A (MCC), carboxylase acetyl-CoA carboxylase, propionyl-CoA carboxylase (PCC), and pyruvate carboxylase (PC) [2]. As a covalently bound coenzyme of carboxylases, it participates in the metabolism of carbohydrates, fatty acids, proteins and nucleic acids [3]. Research shows that a biotin-deficient diet decreases lymphocyte PCC activity and the total protein (TP) expression level of biotin-dependent carboxylases, such as MCC and PCC [4]. Biotin plays an essential role in skin formation, maintenance and repair as a coenzyme for many carboxylases in protein synthesis and fatty acid metabolism [5]. In broiler commercial conditions, more biotin is required to reduce contact dermatitis rather than to only support growth [6]. For fur animals, insufficient biotin intake not only affects animal growth, but also affects skin morphology, hair growth and quality [7]. An *in vitro* study showed that biotin was important in maintaining the viability of isolated sheer secondary hair follicles [5], where supplementation increased the proportion of follicles continuing to grow and biotin deficiency reduced the proliferation of basal keratinocytes [8]. A study from our group showed that supplementation of 10 mg/d rumen-protected biotin (RPB) per goat increased the cashmere growth rate during the cashmere fiber growing period [9]. These results demonstrated potential benefits of biotin and indicated that it may be more pronounced in cashmere goats.

Biotin is broadly distributed in plant feed with lower contents. Due to its wide presence in plants and the microbial synthesis of ruminants, it is generally assumed that biotin deficiency rarely occurs in ruminants. The typical symptoms of biotin deficiency are hair loss, dermatitis, and neuropathy in animals. Distinct signs of acute biotin deficiency are rare in ruminants, while marginal biotin deficiency may has a widespread occurrence. The growth performance and cashmere yield of contemporary cashmere goats is much higher than decades ago, and nutrient requirements of these animals have increased to support higher production, thus supply of biotin of rumen microbial origin may not have kept pace. Studies have demonstrated supplemental biotin exerts beneficial effects on hoof health and integrity [10,11], milk production [12], protein yield and dry matter intake (DMI) [13]. Although studies have evaluated benefits of biotin on production of cows and other animals, few have investigated the biotin requirement in cashmere goats. The need for biotin depends on dietary factors that influence biotin synthesis and degradation [14]. We hypothesized that feeding RPB in the diet of dolings with rapid growth of body and cashmere fibers by correcting an insufficiency in the biotin at the intestinal absorptive site.

The Liaoning cashmere goat is a major cashmere goat breed raised in northeastern China, and is famous for its high net cashmere rate and cashmere yield [15]. Unlike wool and mohair, cashmere growth is seasonal [16]. Given the seasonal growth of cashmere fiber, biotin requirements for cashmere goats are relatively high during the fast growth period of cashmere, that is, from August to November. Therefore, this study sought to investigate the effects of RPB on the growth performance and nutrient digestion and to assess a panel of blood indicators of biotin status in Liaoning cashmere goats during the cashmere fiber growing period, as well as evaluating long-term persistency of their effects. To achieve these aims, we used twin doelings as paired experimental animals to retain the same individual genetic background.

## MATERIALS AND METHODS

All animals used in this study were cared for strictly in accordance with the Chinese Guidelines for Animal Welfare and all procedures were approved by the Institutional Animal Ethics and Welfare Committee of Shenyang Agricultural University (Shenyang, China) under approval number: 201801018.

### Animals, periods, and diets

The study was 16 weeks in length, with two 8-week periods, subsequent to 2 week pre-feeding period. Sixteen twin Liaoning cashmere doelings with an initial body weight (BW) of 24.8±1.20 kg and age of 6 months were used. The twin goats were randomly assigned to two groups. Doelings were housed in individual wooden pens with slotted wooden floors in an open-sided barn and had daily exercise. During weeks 7 and 15, goats resided in metabolism cages for 2 d of adaptation before being placed in sixteen metabolism cages for 5 days.

The basal diet was a complete mixture of 30% concentrate and 70% forage (Table 1). Two groups of animals received the basal diet without (control) or with 10 mg/d per animal of RPB (Beijing Feedig Feed Sci. & Tec. Co., Ltd, Beijing, China). The RPB contained 1% biotin and was produced by microencapsulation technology with D-biotin is covered with multiple layers of coating hydrogenated fat as wall material by freezing spray method. Diets were offered twice daily at 0800 and 1600 hours at ~110% of consumption on the preceding few days after refusals were collected and weighed. All animals had free access to water.

### Measurements

The BW and several linear measures were determined at the beginning, middle, and end of the experiment. The average daily gain (ADG) in each period was determined based on the initial and final BWs. Average DMI (g/d) during the experiment was determined from the actual daily feed intake and calculated based on average BW and BW^0.75^ during the experiment. Linear measures were the length from the point of the shoulder to the hook bone (Hook) and pin bone (Pin), the circumference from heart girth (Heart), the height at the withers (Withers), and the width at the hook bones (Rump). There were 3 body mass index (BMI) calculated as noted below.


$$\begin{array}{l} \text{Hook index}=(\text{Hook}/\text{Withers})\times 100\\ \text{Heart index}=(\text{Heart}/\text{Withers})\times 100\\ \text{Body trunk index}=(\text{Heart}/\text{Hook})\times 100 \end{array}$$

Feed was sampled every two days and biweekly composite samples of feedstuffs were formed. Feed refusals were weighed daily and sampled during the 5-day digestibility period. Total feces and urine collections and sampling was determined for 5 days in 0.6 m×1.2 m metabolism cages in weeks 7 and 15. Feces were collected in plastic-screen baskets placed under the floor of the metabolism cages to keep feces and urine separate, and urine was collected with a funnel draining into plastic buckets containing 10 mL of 10% (vol/vol) of sulfuric acid. Approximately 10% of feces and urine, and approximately 40 g of orts were sampled daily and used to form composite samples for each goat. All samples were stored at −20°C until analysis. Blood was collected by jugular venipuncture into heparinized tubes every 4 weeks in the morning before feeding. Plasma was harvested by centrifugation at 3,000×g and 4°C for 20 min and stored at −20°C until analysis.

### Laboratory analysis

Samples of feed, feed refusals, and feces were ground to pass a 1-mm screen after drying in a forced-air oven at 55°C for 48 h. Samples were analyzed for dry matter (DM), nitrogen, and ether extract (EE) concentration [17]. The neutral detergent fiber and acid detergent fiber were determined using an ANKOM200 Fiber Analyzer (filter bag technique; ANKOM Technology Corp., Macedon, NY, USA), with the addition of a heat-stable α-amylase and sodium sulfite [18]. Plasma TP, urea nitrogen (urea-N), glucose, triglyceride (TG), and total cholesterol (TC) were measured using a spectrophotometer (Ao Yi A360; Shanghai, China) following the instructions of the kit’s manufacturer (Nanjing Jiancheng Bioengineering Institute, Nanjing, China). The concentrations of PCC and methylmalonyl-CoA (M-CoA) were measured by means of the Konelab TM auto analyzer (Thermo Fisher Scientific Oy, Vantaa, Finland), using the enzyme-linked immunosorbent assay (ELISA) test assay kit (Beijing Huaying Biotechnology Research Institute, Beijing, China).

### Statistical analysis

Data were analyzed using a mixed effects model with SPSS 19.0 statistical software (Statistical Package for Social Science; SPSS Inc., Chicago, IL, USA). The fixed effects were treatment, period, and treatment × period, with repeated measures of period and random effect of animal within treatment. Different covariance structures were compared via paired t-test and the statistical significance was set at p<0.05.

## RESULTS

### Animal growth performance

Productive performances, either evaluated at the end of each feeding period or for the entire experimental period, are reported in Table 2. Compared with that of the control group, the ADG of the RPB group increased by 10.94% (p< 0.05), and the DMI increased (p = 0.07). BW (p = 0.321) and the ratio of feed-to gain ratio (F:G) (p = 0.228) did not differ between treatments. Animals during period 2 showed higher ADG (p<0.035) and DMI (p<0.001) than those in period 1. The means of linear measures and BMI of cashmere goats were similar in both groups at initial and finial of a 16 weeks test except initial body trunk index (Table 3), and not affected by dietary treatment (Table 4). Animals during period 2 showed higher linear measures (p<0.05) than those in period 1.

### Intake and digestion during feces collection

For the 5 days when feces were collected, there were some increasing tendencies for the intake of DM and EE (p = 0.070 and 0.070, respectively) and a decrease in the intake of ADF (p = 0.088), and intake of NDF was increased with a p-value of 0.045 (Table 5). However, the digested intake and total tract digestibilities of DM, NDF, ADF, and EE were not affected by dietary treatment (Table 5). The intake of DM, NDF, ADF, and EE was greater for period 2 than for period 1 (p<0.001).

### Nitrogen retention

There were some increasing tendencies for intake (g/d), digestibility (g/d), and digestibility (%) of N (p = 0.062, 0.059, and 0.093, respectively) (Table 6). Fecal excretion of N (g/d) was not affected by dietary treatment, but the percentage of N intake was decreased in the RPB group compared with the control group (p = 0.093). However, urinary N excretion (g/d and percentage of N intake) was not affected by dietary treatment. N retention tended to increase (g/d, p = 0.084; percentage of N intake, p = 0.087) with the addition of RPB to the diet. The total intake of N and fecal excretion of N were greater for period 2 than for period 1 (p<0.001).

### Blood measures

Plasma total protein was increased (p = 0.037) and the urea-N concentration was decreased (p = 0.049) in the RPB diet group compared with the control diet group (Table 7). The concentrations of plasma glucose, TGs, and TC were not affected by dietary treatments. The levels of PCC (p<0.001) and M-CoA (p = 0.013) were increased in RPB group.

## DISCUSSION

Supplementation RPB for 16 weeks increased ADG and the digestible N by 10.94% and 8.5% respectively, compared with the control group. The positive effect of the RPB was probably due to increases in metabolic efficiencies in protein and energy metabolism [14]. For the physiological function of biotin is mainly related to the intermediary metabolism, including a series of carboxylation processes, et., PC, PCC, and M-CoA carboxylase [19]. This is consistent with biotin supplementation research in non-ruminant species, that biotin significantly increased BW losses in laying hens [20]. The weight gain of mink kits also was better in fed a balanced diet with 0.1 mg/kg DM biotin than that of animals fed a low-biotin diet without biotin [7]. These results could be interpreted to that the mechanism of action of supplemental B vitamins is to facilitate increased effciency of metabolic function, and likely be accompanied by increased DMI [21]. Two periods of measurements separated by 8 weeks and a total experiment length of 16 weeks in the present experiment, the lack of interaction between treatment and period in measures of growth performance and others indicating long term effects of RPB over time. The ADG, DMI, and body parameters in period 2 were greater than those in period 1 because of the greater BW and age of the goats.

In studies of this nature in which RPB diets are compared with a control diet, the effects of RPB on digestibility be definitively determined because there are no differences in other characteristics. The apparent total tract DM, NDF, ADF, and EE digestibilities were not affected by biotin supplementation, but N digestibility and retention tended to be improved by RPB. Supplemental biotin had no effect on the apparent total tract DM, organic matter, and NDF digestibilities, which was also reported by Majee et al [13]. Zimmerly and Weiss [12] suggested that biotin may increase milk yield through increased total tract digestibility. The positive N utilization responses to biotin supplementation may be partially due to increased microbial growth or carboxylase activities. However, as there is little information about biotin requirements, dietary supplies, ruminal synthesis and duodenal flows of biotin in ruminants. About effects of biotin supply on nutrients digestive and metabolic efficiency in ruminants still need more research.

In the present experiment, supplementation with biotin increased the plasma total protein and decreased the plasma urea-N levels. Similarly, Girard and Desrochers [22] observed that supplementation with biotin and vitamin B_12_ decreased the release of ammonia across portal-drained viscera and the arterial concentrations of urea-N. Bonomi et al [23] observed no effect of RPB supplements on blood concentrations of urea-N. Absorbed ammonia is mostly from microbial fermentation of dietary protein, although some of it is derived from urea transferred to the lumen of the gastrointestinal tract via blood and saliva or tissue metabolism [24]. In the current experiment, because the intake and digestible N tended to differ between treatments, differences in TP, in spite of an increased removal of urea-N by the gastrointestinal tract, demonstrated that the biotin supplement altered nitrogen metabolism compared with that of goats fed no biotin supplements. This decreased plasma urea-N could be caused by either an increased utilization for protein synthesis or, in contrast, a reduced ruminal degradation of dietary proteins. No direct role of biotin in protein metabolism is known [25].

Similar to most studies [26,27], the present study observed no effect on plasma glucose with biotin supplementation, but some studies have observed an increase [23]. Research has indicated that a large proportion of propionate is metabolized in the rumen wall, where it enters the gluconeogenic pathway, drastically reducing glucose utilization by gastrointestinal tissues and leaving more glucose available for other tissues [22]. Biotin is involved in gluconeogenesis through which amino acids are deaminated for glucose production. In addition to its role in maintaining normal blood glucose concentrations, glucose is utilized as an energy source. Biotin functions in glucose and lipid homeostasis by regulating the expression of genes needed in the regulation of intermediary metabolism [28].

Serum TG and TC concentrations are considered important diagnostic indicators of lipid metabolism. Marshall et al [29] reported a negative correlation between biotin levels and total plasma lipids in healthy individuals. El-Katcha et al [20] also found that blood serum TC and TG were non-significantly altered by biotin use. In accordance with the current experiment, the addition of RPB to the diet had no effect on plasma TP and TC concentrations of cashmere goats compared with goats fed the diet without biotin.

Biotin is a cofactor for microbial enzymes involved in propionic acid synthesis and is also present in carboxylase-containing enzymes that are critical for metabolic roles in mammals. The continuous catalysis of PCC and M-CoA is necessary for propionate entry into the Krebs cycle to provide energy [30]. In the metabolism of amino acids, valine, methionine, isoleucine and threonine, also undergo conversion through the propionyl CoA pathway. Biotin binds to a lysine residue located on the biotin carboxyl carrier protein domain of PCC (the biotin dependent carboxylase enzyme). The lysine-bound biotin moiety is then used to transfer a carbon dioxide molecule to acceptor molecules (i.e., M-CoA) [19]. When the availability of biotin is low, PCC activity is reduced, and conversion of propionyl-CoA to M-CoA is decreased. Midla et al [31] reasoned that biotin supplementation in dairy cows might lead to an increased capacity of biotin-dependent carboxylase enzymes. In this study, RPB supplementation increased the levels of plasma PCC and M-CoA, presumably correlating with enhance the utilization of N the increased of ADG in cashmere goats, but more elaborate studies should be conducted to better understand their dynamic roles in growth performance.

## CONCLUSION

Supplementation of 10 mg/d RPB to the diet increased daily weight gain and plasma total protein, while decreased blood urea-N during the cashmere fiber growing period of cashmere goats. Rumen-protected biotin showed a decreasing trend in the percentage of dietary N excreted in feces, indicating an improved apparent total tract digestibility of dietary crude protein and retention N. Goats with RPB had a higher activity of PCC and M-CoA. Overall, the 10 mg/d RPB to the diet seems to benefit cashmere goats to meet their specific needs.

## Figures and Tables

**Table 1:** Ingredient composition and nutrient levels of the basal diet (dry matter basis)

Item	Content
Ingredients (%)	
Alfalfa	35.0
Peanut straw	35.0
Corn	16.0
Soybean meal	9.5
Fermented soybean meal	3.5
Dicalcium phosphate	0.1
NaCl	0.5
Premix^1)^	0.3
Total	100.0
Nutrient levels^2)^	
ME (MJ/Kg)	9.69
Dry matter (%)	86.99
Crude protein (%)	14.80
Ca (%)	1.00
P (%)	0.32
NDF (%)	47.38
ADF (%)	24.66

ME, metabolizable energy; NDF, neutral detergent fiber; ADF, acid detergent fiber.

1) One kg of premix contained the following: 20 g S, 36 g Fe, 18 g Cu, 19 g Mn, 54 g Zn, 122 mg Co, 168 mg I, 59 mg Se, 1,620,000 IU vitamin A, 324,000 IU vitamin D_3_, 540 IU vitamin E.

2) ME was a calculated value, while others were measured.

**Table 2:** Effects of rumen-protected biotin on growth performance of yearling Liaoning cashmere goats

Item	Treatment^1)^		SEM	Period		SEM	p-values^2)^		
		
	Control	RPB		1	2		Trt	Prd	Trt×Prd
BW (kg)									
Initial	25.70	23.96	0.838				0.076		
Final	34.26	33.46	0.756				0.321		
ADG (g/d)	71.36^b^	79.17^a^	3.090	68.53^b^	82.00^a^	3.090	0.039	0.035	0.272
DMI									
g/d	961.4	1,003.1	15.63	928.3^b^	1,036.1^a^	15.63	0.070	<0.001	0.896
%BW	3.26	3.42	0.150	3.39	3.29	0.150	0.442	0.631	0.611
g/kg BW^0.75^	75.65	79.45	2.647	77.36	77.74	2.647	0.319	0.920	0.754
F:G (g/g)	15.09	13.03	1.551	14.89	13.23	1.551	0.228	0.248	0.166

SEM, standard error of the mean; BW, body weight; ADG, average daily gain; DMI, dry matter intake; F:G, feed-to-gain ratio.

1) RPB, rumen-protected biotin.

2) Trt, treatment; Prd, period.

a,b Means in a row within treatment or period grouping without a common superscript letter are significantly different (p<0.05).

**Table 3:** Means of linear measures and body mass index of yearling Liaoning cashmere goats at initial and finial of a 16 weeks test

Item		Control	RPB^1)^	SEM	p-value
Hook (cm)	Initial	45.38	44.38	1.558	0.542
	Final	52.88	53.38	1.225	0.695
Pin (cm)	Initial	51.38	52.50	2.831	0.703
	Final	60.00	61.75	0.773	0.058
Heart (cm)	Initial	75.00	70.50	2.276	0.089
	Final	80.88	81.63	2.024	0.722
Withers (cm)	Initial	50.00	48.28	1.729	0.345
	Final	57.88	58.13	1.436	0.867
Rump (cm)	Initial	13.56	12.88	0.654	0.328
	Final	15.13	14.88	0.412	0.563
Hook index	Initial	90.81	92.10	0.566	0.622
	Final	91.69	91.93	0.742	0.951
Heart index	Initial	150.3	146.3	0.696	0.419
	Final	140.0	140.5	0.865	0.929
Body trunk index	Initial	165.7^a^	158.9^b^	0.706	0.027
	Final	152.9	153.3	0.240	0.916

RPB, rumen-protected biotin; SEM, standard error of the mean.

a,b Means in a row without a common superscript letter were significantly different (p<0.05).

**Table 4:** Effect of rumen-protected biotin on linear measures and body mass index of yearling Liaoning cashmere goats

Item	Treatment^1)^		SEM	Period		SEM	p-values^2)^		
		
	Control	RPB		1	2		Trt	Prd	Trt×Prd
Hook (cm)	48.00	47.94	0.776	46.72^b^	49.22^a^	0.703	0.955	0.029	0.497
Pin (cm)	55.88	58.03	0.971	55.03^b^	58.88^a^	0.586	0.126	0.009	0.291
Heart (cm)	68.78	66.69	1.343	65.25^b^	70.22^a^	1.149	0.263	0.011	0.307
Withers (cm)	53.66	53.42	0.909	52.11^b^	54.97^a^	0.421	0.815	0.007	0.361
Rump (cm)	14.59	14.06	0.358	13.94^b^	14.72^a^	0.209	0.153	0.039	0.550
Hook index	89.55	89.77	1.625	89.69	89.64	1.163	0.900	0.976	0.919
Heart index	128.2	124.7	2.023	125.2	127.7	1.708	0.177	0.338	0.584
Body trunk index	143.4	139.1	2.142	139.7	142.8	2.708	0.127	0.257	0.456

SEM, standard error of the mean.

1) RPB, rumen-protected biotin.

2) Trt, treatment; Prd, period.

a,b Means in a row within treatment or period grouping without a common superscript letter are significantly different (p<0.05).

**Table 5:** Effects of rumen-protected biotin on intake and digestion by yearling Liaoning cashmere goats during fecal collection

Item	Treatment^1)^		SEM	Period		SEM	p-values^2)^		
		
	Control	RPB		1	2		Trt	Prd	Trt×Prd
DM									
Intake (g/d)	961.4	1,003.1	20.70	928.3^b^	1,036.1^a^	15.63	0.070	<0.001	0.896
Digested (g/d)	778.2	809.9	22.55	750.4^b^	837.7^a^	17.16	0.202	<0.001	0.805
Digestibility (%)	80.78	80.70	0.817	80.67	80.82	0.569	0.920	0.856	0.6114
NDF									
Intake (g/d)	454.5^b^	476.4^a^	9.79	439.9^b^	490.9^a^	7.40	0.045	<0.001	0.905
Digested (g/d)	280.7	298.3	12.63	276.3	302.6	9.40	0.196	0.058	0.870
Digestibility (%)	61.48	62.54	1.647	62.48	61.53	1.172	0.526	0.572	0.759
ADF									
Intake (g/d)	246.9	237.1	5.27	228.7^b^	255.3^a^	3.96	0.088	<0.001	0.747
Digested (g/d)	131.6	130.6	7.26	124.4	137.9	5.75	0.905	0.107	0.989
Digestibility (%)	52.93	55.02	2.244	54.00	53.95	1.725	0.399	0.986	0.953
Ether extract									
Intake (g/d)	11.20	11.68	0.241	10.81^b^	12.07^a^	0.297	0.070	<0.001	0.896
Digested (g/d)	7.29	7.51	0.324	7.07	7.72	0.387	0.515	0.060	0.400
Digestibility (%)	64.79	64.25	1.882	65.09	63.95	1.968	0.753	0.515	0.192

SEM, standard error of the mean; DM, dry matter; NDF, neutral detergent fiber; ADF, acid detergent fiber.

1) RPB, rumen-protected biotin.

2) Trt, treatment; Prd, period.

a,b Means in a row within treatment or period grouping without a common superscript letter are significantly different (p<0.05).

**Table 6:** Effects of rumen-protected biotin on nitrogen utilization by yearling Liaoning cashmere goats during fecal collection

Item	Treatment^1)^		SEM	Period		SEM	p -values^2)^		
		
	Control	RPB		1	2		Trt	Prd	Trt×Prd
N									
Intake (g/d)	22.8	23.8	0.490	22.0^b^	24.5^a^	0.603	0.062	<0.001	0.899
Digestible N (g/d)	14.76	16.01	0.591	14.63^b^	16.15^a^	0.734	0.059	0.023	0.676
Digestibility (%)	64.69	67.28	1.499	66.27	65.70	1.671	0.093	0.705	0.458
Fecal excretion									
N (g/d)	7.99	7.76	0.295	7.36^b^	8.39^a^	0.266	0.379	<0.001	0.199
N/of N intake (%)	35.3	32.7	1.499	33.7	34.3	1.671	0.093	0.705	0.458
Urinary excretion									
N (g/d)	7.50	7.45	0.314	7.23	7.72	0.369	0.881	0.130	0.117
N/of N intake (%)	33.33	31.35	1.622	33.16	31.52	1.859	0.221	0.308	0.134
Retention									
N (g/d)	7.27	8.56	0.673	7.39	8.43	0.824	0.084	0.163	0.744
N/of N intake (%)	31.36	35.93	2.425	33.11	34.18	2.961	0.087	0.679	0.610

SEM, standard error of the mean.

1) RPB, rumen-protected biotin.

2) Trt, treatment; Prd, period.

a,b Means in a row within treatment or period grouping without a common superscript letter are significantly different (p<0.05).

**Table 7:** Effects of rumen-protected biotin on plasma measures of yearling Liaoning cashmere goats

Item	Treatment^1)^		SEM	Period		SEM	p-values^2)^		
		
	Control	RPB		1	2		Trt	Prd	Trt×Prd
TP (gprot/L)	59.90^b^	63.74^a^	1.759	61.74	61.90	1.850	0.037	0.937	0.582
PUN (mmol/L)	6.68^a^	5.98^b^	0.239	6.38	6.28	0.249	0.049	0.777	0.566
GLU (mmol/L)	5.47	5.72	0.222	5.65	5.54	0.275	0.325	0.663	0.679
TG (mmol/L)	1.03	0.98	0.054	0.92	1.08	0.053	0.672	0.168	0.414
TC (mmol/L)	1.92	1.56	0.223	1.76	1.72	0.263	0.186	0.855	0.891
PCC (ng/mL)	27.02^b^	32.55^a^	0.788	29.02	30.55	0.382	<0.001	0.063	0.434
M-CoA (ng/mL)	8.60^b^	9.85^a^	0.474	9.55	8.90	0.781	0.013	0.181	0.960

SEM, standard error of the mean; TP, total protein; PUN, plasma urea nitrogen; GLU, glucose; TG, triglyceride; TC, total cholesterol; PCC, propionyl CoA carboxylase; M-CoA, methylmalonyl CoA.

1) RPB, rumen-protected biotin.

2) Trt, treatment; Prd, period.

a,b Means in a row within treatment or period grouping without a common superscript letter are significantly different (p<0.05).
